# Impact of sex in the prevalence and progression of glioblastomas: the role of gonadal steroid hormones

**DOI:** 10.1186/s13293-021-00372-5

**Published:** 2021-03-22

**Authors:** Claudia Bello-Alvarez, Ignacio Camacho-Arroyo

**Affiliations:** Unidad de Investigación en Reproducción Humana, Instituto Nacional de Perinatología-Facultad de Química, Universidad Nacional Autónoma de México (UNAM), 04510 Ciudad de México, México

**Keywords:** Glioblastoma, Estradiol, Progesterone, Testosterone, Sex differences, Progression, Prevalence

## Abstract

**Background:**

As in other types of cancers, sex is an essential factor in the origin and progression of glioblastomas. Research in the field of endocrinology and cancer suggests that gonadal steroid hormones play an important role in the progression and prevalence of glioblastomas. In the present review, we aim to discuss the actions and mechanism triggered by gonadal steroid hormones in glioblastomas.

**Main body:**

Glioblastoma is the most common malignant primary brain tumor. According to the epidemiological data, glioblastomas are more frequent in men than in women in a 1.6/1 proportion both in children and adults. This evidence, and the knowledge about sex influence over the prevalence of countless diseases, suggest that male gonadal steroid hormones, such as testosterone, promote glioblastomas growth. In contrast, a protective role of female gonadal steroid hormones (estradiol and progesterone) against glioblastomas has been questioned. Several pieces of evidence demonstrate a variety of effects induced by female and male gonadal steroid hormones in glioblastomas. Several studies indicate that pregnancy, a physiological state with the highest progesterone and estradiol levels, accelerates the progression of low-grade astrocytomas to glioblastomas and increases the symptoms associated with these tumors. In vitro studies have demonstrated that progesterone has a dual role in glioblastoma cells: physiological concentrations promote cell proliferation, migration, and invasion while very high doses (out physiological range) reduce cell proliferation and increases cell death.

**Conclusion:**

Gonadal steroid hormones can stimulate the progression of glioblastomas through the increase in proliferation, migration, and invasion. However, the effects mentioned above depend on the concentrations of these hormones and the receptor involved in hormone actions. Estradiol and progesterone can exert promoter or protective effects while the role of testosterone has been always associated to glioblastomas progression.

## Background

**Fig. 1:**
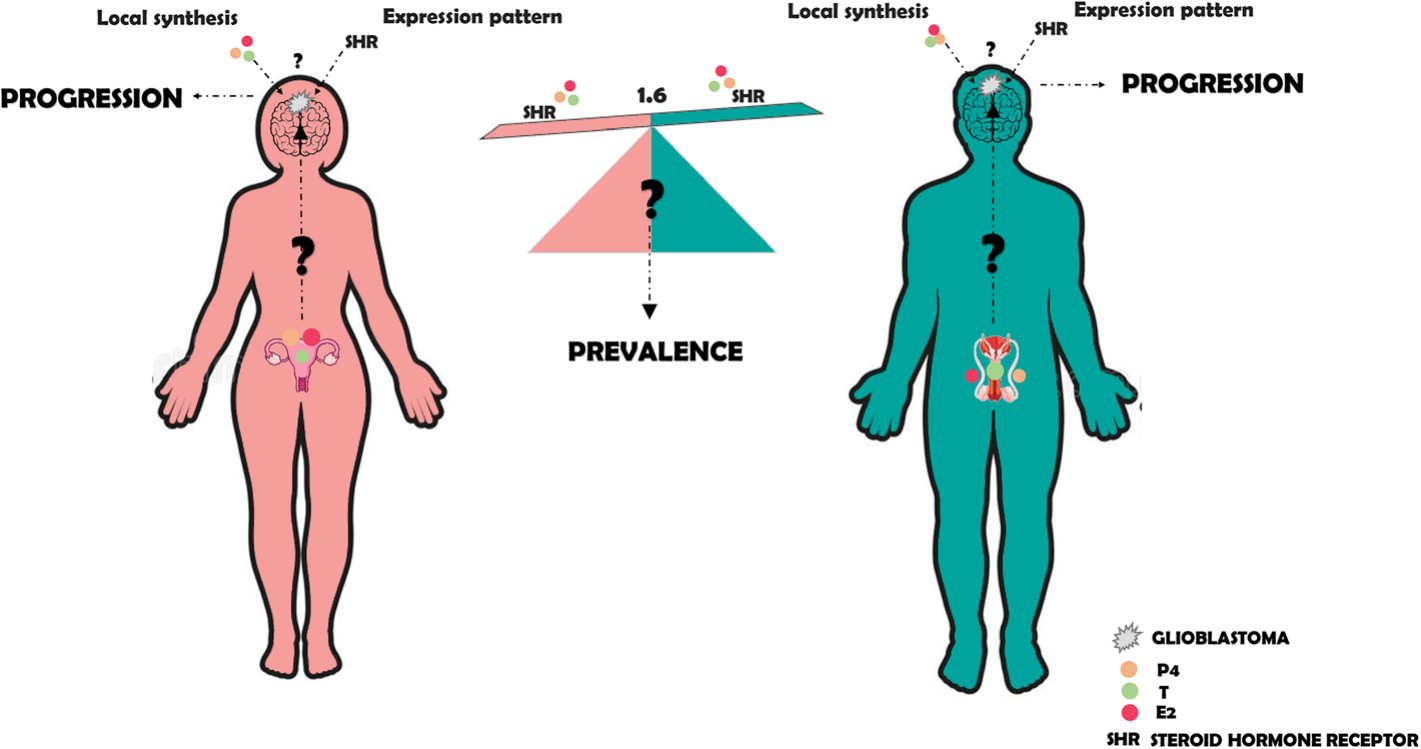
Influence of E2, P4, and T in the prevalence and progression of glioblastoma. Women and men synthesize E2, P4, and T from cholesterol, in the reproductive system, adrenal gland, and brain. Gonadal steroid hormones can also reach the brain by crossing the blood-brain barrier. The evidence accumulated to date suggests that E2, P4, and T from both sources have an important role in glioblastoma progression. However, the significance of E2, P4, and T in the 1.6 males:females prevalence of glioblastoma has not been completely determined. Results obtained nowadays suggest that the role of these hormones in glioblastomas depends on the concentration of hormones and the availability of the isoforms or subtypes of their receptors

Astrocytomas are the most common malignant brain tumors. They are classified according to their malignancy in four grades from I to IV, astrocytoma grade IV, or glioblastoma presents the worst prognostic [1]. Glioblastoma has an average prevalence male-to-female ratio of 1.6/1 (see Fig. 1), and this datum is independent of race, age, economic status, or geographical location [2]. For a long time, the prevalence of glioblastomas in men with respect to women has suggested that sex and specifically, gonadal steroid hormones should participate in glioblastomas growth. Nowadays, a large number of pieces of evidence about the role of these hormones in glioblastomas origin and progression have emerged [3].

Progesterone (P4) and estradiol (E2) are the main female gonadal steroid hormones, while testosterone (T) is the typical male gonadal steroid hormone (see Fig. 1). Besides their classical actions in reproduction, their role in immunological response [4], neuroprotection [5], brain plasticity [6], and cancer are broadly known [7]. The central nervous system (CNS) can synthesize gonadal steroid hormones from cholesterol. Steroids that are synthesized in the CNS are also known as neurosteroids [7]. Recently, the study of neurosteroid functions in the CNS has been extended to brain tumors, mainly glioblastomas. The effects of gonadal steroid hormones over glioblastomas depend on the specific hormone concentration (Table 1) [9], the administration time [10], and the receptor involved in hormone actions [21, 22].

**Table 1:** Effects of low and high concentrations of P4 on glioblastoma cells in in vitro and in vivo models

P4	
Low doses	High doses
*• In vitro effects*	*• In vitro effects*
- Induces proliferation through PR actions (10 nM) (ref [8])	- Decreases cell viability (20–80 μM) (ref [9])
- Increases the expression of EGFR and cyclin D1 through its PR and the recruitment of steroid receptor coactivator-1 (SRC-1) (10 nM) (ref [10])	- Enhances toxicity of TMZ (5 and 80 μM) (ref [11])
- Induces the expression of progesterone-induced blocking factor (PIBF) (10 nM) (ref [12])	- Changes in detoxification mechanisms, stress, immune response, and glucose metabolism (100 and 300 μM) (ref [13])
- Stimulates the unphosphorylated state of cofilin (10 nM) (ref [14])	- Reduction of glycolytic metabolism by decreasing the Glut1 expression (8 and 100 μM) (ref [15])
- Induces migration and invasion through PR actions (10 nM) (ref [14])	
- Increases the number of glioma stem cells from U251 cells (10 nM) (ref [16])	
- Allopregnanolone and 5alpha-dihydroprogesterone (P4 metabolites) increase the number of glioblastoma cells (10 nM) (refs [17, 18])	
- 5alpha-dihydroprogesterone increases the migration of glioblastoma cells (10 nM) (ref [18])	
*• In vivo effects*	*• In vivo effects*
- Increases the area and infiltration of tumor through PR actions (1 mg) (refs [8, 19, 20])	- Reduction of tumor volume (8 and 100 μM) (ref [9]) (100 and 200 μM) (ref [15])
	- Induction of cell senescence by attenuating the signaling pathway PI3K/Akt/mTOR (8 and 100 μM) (ref [9])

For these reasons, the precise role of this kind of hormone in glioblastomas is controversial. Besides, the intrinsic molecular, cellular, and tissue differences of each sex also impact the prevalence and progression of glioblastomas.

Even when receiving the standard therapy, consisting of surgical resection followed by radiotherapy and chemotherapy with temozolomide (TMZ), patients with glioblastoma relapse in a period not much longer than a year. This therapy has remained without changes for more than a decade [23]. Compile and analyze the evidence about the actions of gonadal steroid hormones in glioblastomas is an essential step in the understanding of the whole picture of this complex disease. According to accumulated knowledge until the present, gonadal steroid hormones exert a variety of important actions in glioblastoma progression through the promotion of proliferation, migration, and invasion. Considering these facts, in this review, we discussed the influence of these steroids in the prevalence and progression of glioblastomas in the context of the molecular differences between sexes.

## Influences of sex differences in cancers of non-reproductive tissues

Gonadal steroid hormones have a high impact on the incidence and evolution of several cancers in males and females. Sex hormone levels are different between them, and therefore their role in pathophysiological processes. However, sex hormones do not constitute the unique factor influencing the differential prevalence and course of cancer in males and females. Recently, the intrinsic genetic conditions have gained great relevance. Most important is the fact that besides sex chromosomes, the autosomal genes also contribute to this heterogeneity. The regulation of the autosomal genome is sexually dimorphic [24]. Reinius and cols found hundreds of genes with sex-biased expression patterns in the brain cortex of male and female humans and macaques [25].

Interestingly, the brain cortex is the main localization site of glioblastomas. The differences in gene expression patterns between sexes have a significant influence on predisposition to develop certain diseases. Several cancer types are more frequent and have a worse prognosis in men than in women [26]. According to Global Cancers Statistics, colon, rectum, stomach, liver, esophagus, bladder, skin, pancreas, Kaposi sarcoma, lip, and oral cavity cancers have a higher incidence in men than in women. In colorectal cancers, it has been reported that estrogens play a protective role against colorectal cancer development in female mice [27]. In contrast, testosterone has the opposite effect in male rats [28]. Nevertheless, the differences in the prevalence of colorectal cancer between both sexes are present both in children and adults. The last idea suggests that other factors besides gonadal steroid hormones influence the prevalence and prognosis of cancer between females and males. In kidney cancer, one potential explanation for the higher prevalence in men is the X chromosome-encoded mutations since these are more frequently found in tumors derived from males. Specifically, the mutation in the KDM5C gene has a significant impact on tumorigenesis [29]. Besides, other authors have reported that gonadal steroid hormones also exert effects on kidney cancer cells. E2 inhibited the growth of renal cell carcinoma in an estrogen receptor (ER)-dependent pathway [30], and on the contrary, dihydrotestosterone (DHT) promoted renal carcinoma growth [31] via androgen receptors. Gastric cancer is more frequent in men than in women in a 2:1 proportion. As in the previous examples in this type of cancer, E2 plays a protective role since it induced apoptosis at different concentrations by activating caspase 3 and inhibiting Bcl-2 and Bcl-xL [32]. Interestingly, in gastric cancer, the protective role of E2 depends on the activated ER subtype [33]. One of the few examples regarding a higher prevalence in women than in men (2.9 times) is thyroid cancer. It has been suggested that E2 induces ERα expression over ERβ, promoting proliferation and growth [34]. Some of the tissues previously mentioned can synthesize gonadal steroid hormones, which have an essential role in maintaining cellular homeostasis. For example, in the intestine, estrogens are necessary to preserve the epithelial barriers and reduce the permeability. However, other organs such as kidney express ERs but it cannot synthesize gonadal steroid hormones from cholesterol [35].

Yuan and cols analyzed the molecular differences between male and female patients in 13 cancer types, including glioblastomas. They found two sex-effect groups with different profiles according to the prevalence and mortality. The weak sex-effect group contained few sex-biased genes associated with prevalence and mortality ratios more similar between females and males. In contrast, the strong sex-effect group contained a greater number of molecular signatures influenced by sex and more separated ratios of prevalence and mortality between females and males. In this study, glioblastomas were located in weak sex-effect groups containing few sex-biased genes [26]. As the previous types of cancers discussed, brain tumors including glioblastomas are susceptible to gonadal steroid hormones action. It is also important considering the ability of CNS to synthesize gonadal steroid hormones from cholesterol and that these hormones can regulate the expression of several genes. In the brain, gonadal steroid hormones display a variety of actions that conduce to brain plasticity [6], for example in the amygdala and hypothalamus of fetal rats, E2 promotes neurogenesis through the increase in proliferation and survival of new neurons [36]. Neuroprotective effects of E2 have been demonstrated in primary cultures of hippocampal neurons since this hormone prevented neuronal death induced by glucose deprivation [37]. In orchidectomized rodent males, T or DHT increases spine dendritic density [38]. In primary cultures of rat cortical neurons, P4 increases the phosphorylation of a group of proteins involucrate in cytoskeleton remodeling, such as focal adhesion kinase and Wiskott-Aldrich syndrome protein family member 1 [39]. It has been observed that P4 regulates sexual behavior increasing the lordosis through the PR-B isoform in females rats [40]. In the case of glioblastomas, it has been reported that P4 and E2 increased the expression of proteins closely associated with angiogenesis and cell proliferation, such as vascular endothelial growth factor (VEGF), epidermal growth factor receptor (EGFR), and Cyclin D1 in human glioblastoma derived cell lines. In the case of E2, this effect was induced through ERα [10, 22].

## Prevalence of brain tumors in female and male patients

In 15 countries on six continents, it has been reported an evident prevalence of brain tumors in males in comparison with females ranged from > 1 to 3.5 for astrocytomas (including glioblastomas), medulloblastomas, ependymomas, and oligodendrogliomas [41]. Neuroblastomas, the most common extracranial solid tumors in children, originated from neural crest cells, have a modest difference in the prevalence between males and females (boys:girls ratio = 1.2) [42]. The prevalence of vestibular schwannomas, benign and slow-growing brain tumors, depends on age. Between 35 and 54 years, the incidence is higher in females, whereas between 65 and 84 years, the incidence is higher in males [43]. Chordomas, tumors of the sarcoma family that occur midline along the spinal axis, affect men more commonly than women in an approximately 2:1 ratio [44]. In medulloblastomas and glioblastomas, the male predominance is evident both in the pediatric and adult populations [45]. This suggests that other factors besides gonadal steroid hormones are involved in the prevalence of brain tumors.

The dysregulation of the MAPK pathway is a frequent mechanism involved in the over-proliferation of many cancer types such as astrocytomas. In some areas of the male mice brain, it has been observed a higher activation of the MAPK pathway, independent of gonadal steroid hormone status [46]. On the contrary, in vitro studies have shown a greater activation of the MAPK pathway in female astrocytes. However, when female astrocytes were treated with E2, the inhibition effect over the MAPK pathway was more potent than in male astrocytes. Furthermore, in female astrocytes, the effect of E2 triggers a higher apoptosis rate [47]. This latter effect in females could be associated with ER’s different expression patterns between males and females [48]. This evidence is insufficient to explain the differences between females and males in the prevalence and progression of brain tumors. However, considering E2 and its receptors are involved in the regulation of MAPKs signaling, this hormone could play an essential role in the prevalence and progression of astrocytomas through its interaction with this pathway.

## Glioblastoma

Glioblastoma or astrocytoma grade IV is the most common malignant primary brain tumor, representing 56% of all gliomas. Patients with glioblastoma have an extremely poor prognosis with overall survival of 15 months, and to date, there is no effective therapy for the treatment of this malignancy. The standard gold therapy for glioblastoma has been unmodified for more than ten years [49]. Epidemiological data refers that primary glioblastomas are more common in men (1.6 males:females ratio) while secondary glioblastomas (evolve from low-grade astrocytoma) appear more frequently in women (0.65 males:females ratio) [50]. The higher incidence of primary glioblastomas in men suggests a potential inductor role of male gonadal steroid hormones in the occurrence of these tumors. In secondary glioblastomas, these data suggest that female gonadal steroid hormones are related to the progression (from low-grade astrocytomas) rather than the prevalence. Other factors besides gonadal steroid hormones, such as intrinsic genetic and molecular differences, have been linked to glioblastoma prevalence in the male population.

### Intrinsic genetic and molecular differences in the prevalence and progression of glioblastomas

The contribution of sex chromosomes to sex differences has long been recognized. The differences in the dosage of X-linked genes have been associated with the sex-specific genetic architecture of some diseases [24], such as dyskeratosis congenita [51], and severe combined immunodeficiency syndromes [52]. Dunfords and cols provided pieces of evidence to support the EXITS theory. Some tumor suppressor genes (TSGs) in X-chromosome escape from X inactivation; these genes are known as EXITS genes for “escape from X-inactivation tumor suppressors.” These authors suggest that mutations in TSGs that escape X-inactivation have a significant influence on a male predominance of cancer, or in other words, that biallelic expression of EXITS genes confers a protection status against cancer in women. In the specific case of glioblastomas, these authors found proof of biallelic expression in females of KDM6A (encodes the lysine-specific demethylase 6A), KDM5C (encodes the lysine demethylase 5C), DDX3X (encodes a DEAD-box helicase 3 X-linked), and ATRX (encodes the ATRX chromatin remodeler). A higher expression of these genes was detected in females with respect to males [53]. These results are just a proof of clear intrinsic genetic differences between men and women with glioblastoma. However, the functional role of these genes in glioblastomas prevalence has not been demonstrated. The transition to the persistent state of glioblastoma stem cell (GSC) is dependent on the upregulation and widespread redistribution of histone demethylases KDM6A/B [54]. Sun and cols went a little further and found that male astrocytes from mesenchymal glioblastoma carried out a greater inactivation of tumor suppressor RB than female astrocytes [55]. Also, these authors questioned the basis of difference in the RB regulation between males and females, and they found that female astrocytes respond with greater activation of the cyclin–cyclin-dependent kinase (CDK) complexes inhibitors p16 and p21 against serum deprivation or the potent antineoplastic etoposide than male astrocytes. These results were obtained under conditions that promoted the growth arrest dependent on Rb; thereby, the difference in the status of p16 and p21 is a critical element in the higher prevalence of glioblastomas in males [56].

### Gonadal steroid hormones in the prevalence and progression of glioblastomas: E2

**Fig. 2:**
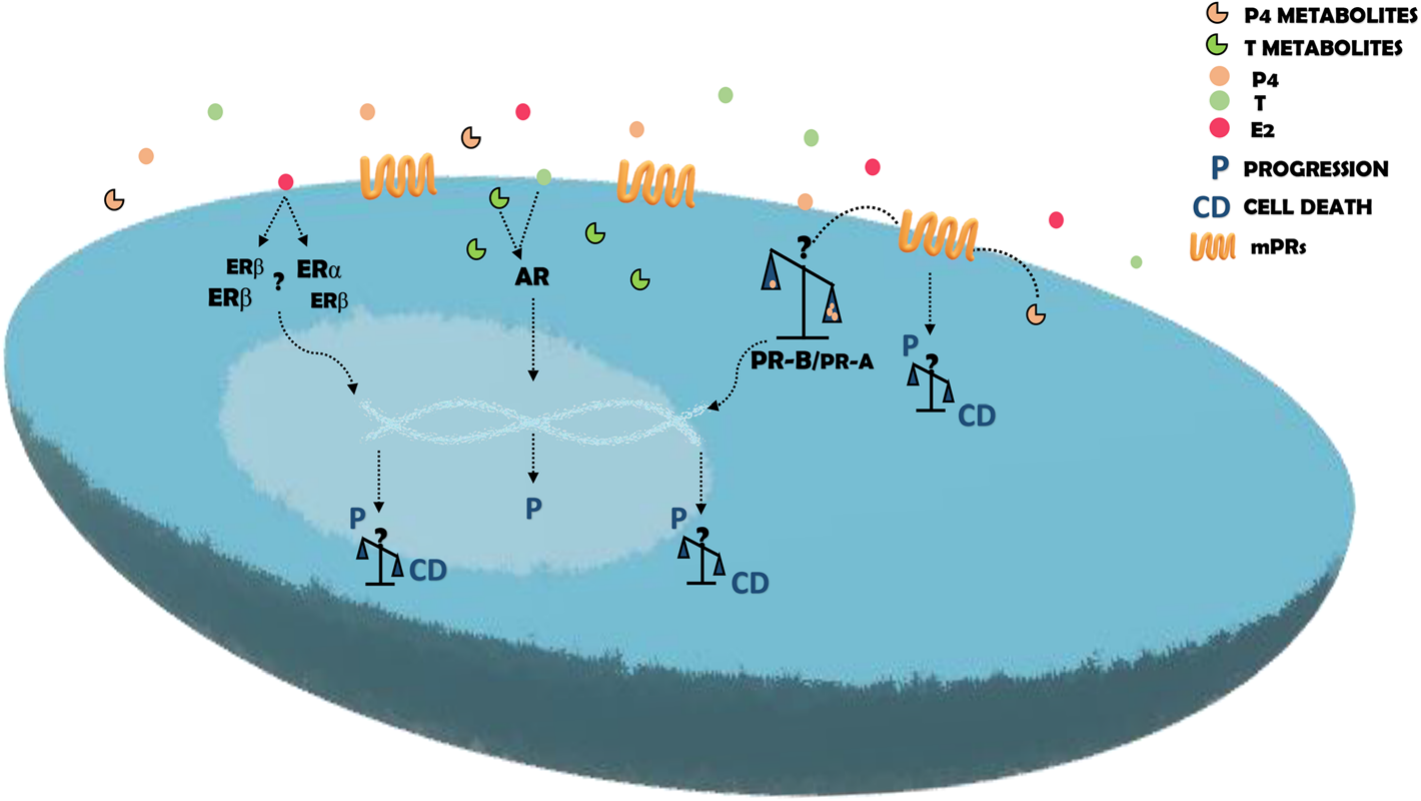
Molecular mechanisms of E2, P4, and T action in glioblastoma cells. Once in glioblastoma cells, E2, P4, and T can activate intracellular o membrane receptors, and according to the cellular context, different effects will be triggered. Effects induced by E2 are mainly mediated by the ERα/ERβ ratio. In a context with an ERα prevalence, E2 is associated with glioblastoma progression through enhancing proliferation, EMT, migration, and invasion, while ERβ prevalence is associated with cell death. In the case of P4 and its metabolites, a dual behavior is associated with the concentration of the former. Low levels of P4 induce an increase in proliferation, migration, and invasion while high levels induce processes related to cell death. P4 exerts its action through the PR or mPRs. The effects of T and its metabolites have been always associated with events that promote the progression of glioblastomas

Based on the average male-to-female ratio of 1.6/1 for glioblastomas, female gonadal steroid hormones have been considered as possible factor protection against them [57]. However, this hypothesis excludes secondary glioblastomas, which are more frequent in females than in males [50]. Nuclear steroid receptors play an essential role in the actions of gonadal steroid hormones. E2 can induce various effects through its classical receptors, ERα, and ERβ (see Fig. 2). In the glioblastoma context, results obtained about this hormone’s role are heterogeneous and depend on the ER subtypes expression. Batistatou and cols in 2004 found that the expression of ERβ proportionally decreases according to the grade of malignancy of astrocytomas. Two years later, the same authors found that the low expression of ERβ was correlated with the worst survival of patients with astrocytomas [58, 59]. Sareddy and cols found similar results in terms of the decrease of ERβ expression with glial tumors' progression [60]. However, another group (Li and cols) found that ERβ5 is the isoform predominant in human gliomas, and its expression increase with the malignancy grade [61].

Recently, Hernández-Vega and cols found a slight but significant increase in the mRNA levels of ERα and ERβ in 155 glioblastomas compared to 167 low-grade gliomas. It was also determined that the mRNA expression of ERα and ERβ was higher in the mesenchymal glioblastoma subtype compared to the other three subtypes defined by Verhaak and cols [62]. Besides, using TCGA analysis, a higher expression of both ER subtypes was associated with a poor clinical outcome [63]. On the contrary, Jimenez and cols found that the lowest expression of ERα mRNA was associated with a bad prognosis [64]. Hönikl and cols, through the analysis of 60 tissue samples, determined that the high expression of ERα and aromatase was correlated with a significantly higher survival probability of GBM patients, regardless of gender [65]. González-Arenas and cols demonstrated that estrogens can induce the growth of human astrocytomas through the ERα and by recruitment of coactivators SRC-1 and SRC-3 [22]. In contrast, the ERβ agonist DPN inhibited the cell proliferation of glioblastoma-derived cell lines T98G, U87, LN229, and U138 and stimulated cell death in a xenograft model in mice [60]. Despite the contradiction related to the value of ERα and ERβ as a prognostic factor and their expression in gliomas of different malignant grade, these results suggest that E2 has a protective role against gliomas through its ERβ; however, this effect may depend on the amount of each ERβ isoform (see Fig. 2). In breast cancer cells, the same dual behavior of E2 through ERα and ERβ has been found [66].

It would be interesting to establish a relationship between levels of ER subtypes in males and females and their influence over the regulation of p16 and p21 effects in each sex.

An interesting data obtained by Wei Yang and cols suggested that male glioblastoma patients could have a better response to inhibitors of the cell cycle. In contrast, female patients would do better with integrin signaling inhibitors [67]. According to Sun and cols, this hypothesis is based on results who found that female astrocytes have a stronger antiproliferative response than male astrocytes by the p16 and p21 activation [55]. Considering that integrins are essential to change the cell interactions with the ECM, one of the steps to epithelial-mesenchymal transition, based on Yang’s results and the last pieces of evidence exposed, it could be assumed that E2 plays a role in the epithelial-mesenchymal transition of glioblastomas. Importantly, Hernández-Vega and cols have recently demonstrated that E2 induced the epithelial-mesenchymal transition in human glioblastoma cells through the activation of ERα. These authors found that E2 and PPT, an agonist of ERα, modified glioblastoma cell morphology, increased cell migration, and the expression of EMT markers such as vimentin N-cadherin. At the same time, the treatment with MPP, an antagonist of ERα, blocked the E2 and PPT effects. The agonist of ERβ, DNP, did not affect any processes [63].

After these pieces of evidence, the role of E2 in the prevalence or progression of glioblastoma is still controversial since it seems that E2 actions in glioblastomas depend on cell context (see Figs. 1 and 2).

### Gonadal steroid hormones in prevalence and progression in glioblastoma: P4

The most studied hormone in the context of glioblastomas is P4. Several pieces of evidence have implicated this hormone and its reduced metabolites in the progression of glioblastomas. Since 1997, some studies have related the progesterone receptor (PR) content with astrocytomas malignancy. For example, Khalid and cols found that PR was more expressed in glioblastomas (astrocytoma grade IV) than in astrocytomas grade I and II from biopsies of 86 patients [68]. Also, González-Agüero and cols found that PR content at the protein level was observed in 83% of astrocytomas grade III biopsies while 100% of glioblastoma biopsies were positive for PR signal. These authors also found that the predominant isoform in the biopsies analyzed was PR-B over PR-A (see Fig. 2) [69].

Interestingly, there exist some pieces of evidence about the behavior of astrocytomas during pregnancy. This period is characterized by the highest increase in P4 levels, up to 200-fold. In 2016, Hanada and cols reported the quickest ever progression of diffuse astrocytoma during pregnancy. A 21-year-old woman was diagnosed with a low-grade astrocytoma during her first pregnancy. In the second pregnancy, at the 4th month, the biopsy revealed the presence of diffuse astrocytoma, and 2 months later, the tumor was surgically removed. The histological diagnostic confirmed a glioblastoma [70]. In 2018, Peeters and cols carried out a multi-institutional retrospective study with 50 pregnant women diagnosed with a glioma. Of 24 women who had been diagnosed with a glioma before pregnancy, 87% of the cases showed an increase in the growth rate of the tumor, and 38% of the cases showed a clinical deterioration with seizures that were only resolved after delivery. In the case of 28 women diagnosed with glioma during pregnancy, the tumors were discovered during the second (29%) and the third (54%) trimesters. In 21.4% of the cases, the clinical deterioration improved after delivery. These authors concluded that pregnancy can unfavorably impact over glioma progression [71], and parity attenuates this effect. These facts are in line with the results of in vitro assays detailed below.

To understand the role of PR in glioblastomas progression, some authors have carried out functional assays by stimulating or inhibiting PR. González-Agüero and cols treated cell lines derived from human glioblastomas with P4 at 10 nM (Table 1). They observed a significant increase in the proliferation rate of these cells compared to the vehicle. When these authors used RU486, an antagonist of PR, the latter’s effect was blocked, which suggests that P4 induces the proliferation of glioblastoma cells through its PR [8]. These findings suggest a potential role of P4 via its intracellular receptor in the progression and malignancy of astrocytomas. However, these pieces of evidence do not correspond to the supposed protective role of female hormones in glioblastomas. Previously, this review mentioned the importance of the specific concentration of hormones. Regarding this fact, it is essential to cite a group of authors, Atif and cols, who found a dual behavior in the effects induced by P4 depending on the concentration. In the proliferation assays conducted by these authors, cell lines derived from glioblastomas showed an increase in viability at physiological concentrations, as the Camacho-Arroyo group has reported. In contrast, at very high concentrations (Table 1), P4 decreased cell viability [9]. Besides the antiproliferative effects induced by P4 at high concentrations, it has been found that P4 enhances the cytotoxic effects of temozolomide in glioblastoma cells and reduces its toxicity in normal cells [11]. Antiproliferative effects of high concentrations of P4 have been related to the reduction of glycolytic metabolism and the induction of cell senescence by decreasing the Glut1 expression and attenuating the signaling pathway PI3K/Akt/mTOR [15]. A recent proteomic analysis, conducted by Altinoz and cols suggests that suppressive actions of high doses of P4 on glioblastoma are induced by changes in detoxification mechanisms, stress, immune response, and glucose metabolism [13]. High doses of medroxyprogesterone acetate, a synthetic variant of P4, have also shown antiproliferative effects on glioblastoma-derived cell lines such as C6 and U87 [72].

The action of P4 over the proliferation of glioblastoma cells has also been demonstrated in in vivo models. In this case, P4 increased the area of tumor derived from human astrocytoma grade III cells that were implanted in the motor cortex of male rats. This effect was blocked when RU486 was administrated together with P4 [19]. Similar results were obtained when U87 human glioblastoma-derived cell line was implanted in the cerebral cortex of rats, and phosphorothioated antisense oligodeoxynucleotides (ODNs) against PR expression was used [20].

P4 also promoted the migration and invasion of U251 and U87 human glioblastoma-derived cell lines, and when RU486 or oligonucleotide antisense against PR were used together with P4, the effect of P4 was blocked. P4 also stimulated the unphosphorylated state of cofilin, which is fundamental in the actin cytoskeleton remodeling [14]. In 2014 Germán-Castelán and cols implanted U373 cells in Wistar adult male rats’ motor cortex and observed that in rats treated with P4, the tumor grew and infiltrated to deeper structures of the brain in a higher proportion compared to vehicle [19].

According to its capacity to stimulate proliferation in glioblastoma cells, P4 also promotes the overexpression of some genes closely related to molecular proliferation pathways. In human glioblastoma derive cells, P4 increased the expression of EGFR and cyclin D1 through its PR and the recruitment of steroid receptor coactivator-1 (SRC-1) [10].

Another evidence about the role of P4 in the proliferation of glioblastoma cells is related to the capacity of these hormones to induce the expression of progesterone-induced blocking factor (PIBF) [12]. This protein has been associated with the proliferative and immunologic effects of P4 in some malignancies [73]. PIBF has been associated with the suppression of anti-tumor immunity in a mode like those used by the embryo during pregnancy [74]. Kyurkchiev and cols found that PIBF was intracellularly expressed by primary culture cells derived from six samples of glioblastoma patients. Considering the immunosuppressant role of PIBF, these authors suggested that glioblastoma cells escape from the immune system by expressing this factor [75]. In cells derived from human glioblastoma, P4 induces the increase of the expression of PIBF, which in turn leads to the increase of the number of cells and in the JAK1 and STAT6 phosphorylation at 20 min [76].

Therefore, in glioblastoma cells, P4 can modulate the immune and growth response through increased PIBF expression.

Not only P4 but also its metabolites such as allopregnanolone and 5alpha-dihydroprogesterone (DHP) can induce an increase in the proliferation of glioblastoma cells. Zamora-Sánchez CJ and cols found that allopregnanolone and 5alpha-dihydroprogesterone, both reduced metabolites from P4, increased the number of U87 and U251 cells, similarly to P4 [17, 18]. Besides, DHP increased the migration of glioblastoma cells. These effects reinforce the role of P4 over the growth of glioma cells.

Standard care treatment for glioblastomas provides only an overall survival of 15 months. The poor benefices of the standard gold therapy (maximal safe surgical resection followed by combined chemotherapy with temozolomide and radiation) for glioblastomas are attributed to cancer stem cells, which provide the tumor the capacity of invasion, resistance to the therapy, and recurrence. In this context, Piña-Medina and cols demonstrated that PR expression is higher in glioma stem cells (GSCs) than in glioblastoma cell lines and that P4 was able to increase the number GSCs from U251 cells [16]. Chek and cols found that a 43-year-old male with glioblastoma multiforme exclusively treated with RU486 showed a significant improvement of quality of life since the patient improved speech and movement of his hands after 2 weeks of starting the treatment with RU486 [77].

PR is not the only receptor involved in P4 effects in glioblastoma cells. P4 can also interact with membrane progesterone receptors (mPRs) (see Fig. 2) and the progesterone receptor membrane component-1 and 2 (PGRMC1-2). In immortalized granulosa cells, the depletion of PGRMC1 and PGRMC2 attenuated some actions exerted by P4 [78]. P4 decreased PGRMC1 mRNA content in LN-229 spheroids, while in U87 spheroids, P4 increased the protein content of the PGRMC1 [79]. mPRs are G protein-coupled receptors members of the progestin and adipoQ receptor (PAQR) Family and five subtypes have been described (mPRα, mPRβ, mPRγ, mPRδ, and mPRε) [80]. In the context of glioblastoma, expression of mPRα, mPRβ, mPRγ [81], mPRδ, and mPRε has been detected in U87 and U251 cells. The analysis of expression data from TCGA revealed that mPRβ, mPRδ, and mPRε were downregulated in GBM compared to normal tissues. mPRδ expression was negatively correlated to the tumor grade, while mPRε expression was independent of the tumor grade. The analysis of the clinical outcome of both mPRs showed that the low expression of mPRδ was correlated to poor prognosis. At the same time, the contrary behavior was observed in the case of mPRε [82]. In U87 and U251 cells, Org OD 02-0, a specific mPRα agonist, increased cell proliferation, migration, and invasion. The addition of siRNA against mPRα blocked the agonist effects [83].

Until this point, it seems very clear the potential role of P4 (at low concentration) (Table 1) and its metabolites to promote the progression of glioblastoma cells by interacting with PR or mPRs (see Fig. 2).

### Sex steroid effects on GBMs are derived from hormones synthetized in the brain or in the endocrine glands?

Neurons, glial cells [84, 85], and glioblastoma-derived cell lines such as U87 and C6 [86] can synthesize neurosteroids from cholesterol. Besides, gonadal steroid hormones can also reach the brain. The effect of these hormones does not depend on their origin in the organism, but on the concentration and the receptor subtype they interact with. For this reason, it is difficult to relate the effects of E2, P4, and T on glioblastomas with the organ they were synthesized. In the hippocampus, for example, the concentration of estradiol (E2) is sixfold higher than in plasma [87]. In this brain region, E2 plays an essential role in brain plasticity through the induction of proliferation and the increase in the frequency of multiple synapse boutons in CA1 neurons [88]. If we consider the concentration of E2 in this area compared to that in plasma, the most logical thought is to attribute the effects described above to E2 synthesized in the hippocampus. In contrast, in the subventricular zone, the levels of circulating testosterone (T) are fundamental to induce proliferation, since the subventricular zone of castrated rats exhibited less 5-Bromo-20-deoxyuridine (BrdU) incorporation than control or castrated animals treated with T [89]. Then, at least in events related to brain plasticity, the specific brain region is an important factor to consider. In the case of glioblastomas, there is no information about differences in gonadal steroid hormones concentration among plasma, tumor, and tumor microenvironment; however, in one study conducted by Bao and cols, serum levels of T were higher in patients with glioblastoma than in non-cancer patients [90]. A pilot study with 36 biopsies of astrocytoma patients revealed that E2 concentration was higher in astrocytomas grade IV (glioblastomas) than in grade II or III astrocytomas. Besides, the highest expression of aromatase was associated with the worst survival prognostic in glioblastoma patients [64]. In another study by Plunkett and cols, in which nude rats received intracerebral implants of U87MG cells, ovariectomized female rats died first than sham-ovariectomized animals [91], which suggest the relevance of gonadal steroid hormones in glioblastoma progression. Taking into consideration these pieces of evidence, we consider that gonadal steroid hormones and neurosteroids have an essential role in the effects discussed in this review.

### Risk of glioblastoma associated to exogenous female gonadal steroid hormones

Up to this point, we have discussed the role of endogenous E2 and P4 in the prevalence and progression of glioblastomas. However, several studies have reported the significance of exogenous female gonadal steroid hormones, such as the administered as contraceptives or hormone replacement therapies, and the risk of developing a glioblastoma. In 2006, Wigertz and cols studied hormonal contraceptives and hormone replacement therapy on the glioma risk. These authors included 115 glioma cases and 323 controls; however, their results did not show any correlation between hormone usage and glioma risk [92]. Over the years, this result has been contradicted by other authors. In 2011, Cowppli-Bony and cols collected the available information from 20 articles with data related to the influence of exogenous female gonadal steroid hormones on the risk of developing a glioma. The analysis showed that replacement therapy and oral contraceptives were associated with a reduced glioma risk [93]. In another analysis, which incorporated 11 studies with 4860 cases and 14,740 controls, a lower risk of glioma was observed in women who were ever users of oral contraceptive and hormone replacement therapy than those who had never used them [94]. Anic and cols carried out a case-control study of 507 glioma cases, 247 meningioma cases, and 695 community-based controls. These authors found that glioma cases were less likely to be presented in women who used long-term oral contraceptives [95]. However, another study conducted by Andersen and cols showed that the use of hormonal contraceptives for an extended period increases glioma risk. This study was performed using Denmark’s national administrative and health registries with 317 glioma cases and 2126 controls [96]. Most recently, in 2018, a meta-analysis of case-control and cohort studies showed that a decreased risk of gliomas is associated with the use of hormonal contraceptives and hormone replacement therapy. Despite the contradictions, the evidence suggests that the use of exogenous female gonadal steroid hormones decreases the probability of developing a glioma [97], which supports the idea of female gonadal steroid hormones' protection against glioma development. However, it is essential to note that the most used contraceptives combine estrogens and progestins, then the effects of this therapy are caused by this combination, and not by only estrogens or only progestins. González-Arenas and cols found that tibolone, a selective tissue estrogenic activity regulator, commonly used in the treatment of menopausal symptoms, increased the proliferation of U251 and U87 derived glioblastoma cells lines, and this effect was blocked by ERs or PRs antagonists, ICI 182, 780, and RU 486 [98].

### Gonadal steroid hormones in prevalence and progression in glioblastoma: T

Regarding the role of male gonadal steroid hormones, the first study about the relationship between T levels and glioblastoma was conducted in 2017 by Bao and cols. These authors found that T’s levels in the serum of glioma patients were higher than in the control group and the benign brain tumor group. However, the levels of T in the serum did not change among other astrocytomas WHO grades. In this study, the authors also discovered that the androgen receptor (AR) promoted the proliferation of cell lines derived from human glioblastoma through suppressing p53 [90]. There are also no data on the difference in AR levels between men and women in glioblastomas, but in animal models [48] and humans [99, 100], the content of AR is higher in a variety of male brain areas compared with the female brain. More than two decades ago, Brentani and cols detected AR’s expression in 42% of 12 glioblastoma samples [101]. Paoletti and cols found the expression of AR in 21.6% of 57 samples from 25 glioblastomas, 18 anaplastic astrocytomas, and 14 other types of astrocytomas [102]. Over the years, other authors have found similar results regarding AR in glioblastoma cells [103–105]. In 2014, Liu and cols compared the expression of AR between high-grade astrocytic tissue and low-grade astrocytic tissue, and the levels of AR were significantly lower in high grade astrocytic compared to low-grade astrocytic tissue, and the expression of this receptor was negatively correlated to the differentiation of astrocytic tumors [106]. Yu and cols found a higher AR expression in 22 samples from male GBM patients than the expression in the normal periphery brain. These authors also detected the AR in eight human GBM cell lines: A172, LN-18, LN-229, M059, T-98G, U87-MG, U118-MG, and U138-MG. In this study, the authors discovered that DHT antagonized the cell growth induced by TGFβ and increased the apoptosis rate [107]. However, in 2018, another group found that AR antagonists induced cell death in T98G, U87MG, and A172 cell lines [108]. A more extensive study carried out by Rodriguez-Lozano and cols found that T induced the proliferation, migration, and invasion of U251 and U87 cells. These effects were blocked when flutamide, an antagonist of AR, was used [109]. T is principally metabolized to DHT by the enzyme 5α-reductase (5αR). In cell lines derived from human glioblastomas, this metabolite increased the proliferation, migration, and invasion, whereas the treatment with finasteride and dutasteride, both inhibitors of 5αR, blocked the effects induced by T, which means that T promotes the previous effects in glioblastoma cells through its metabolite DHT. In this study, the TCGA Data Analysis of mRNA expression of AR, 5αR1, and 5αR2 from 196 grade II, 223 grade III, and 139 grades IV were compared with 249 healthy brain cortex samples. This analysis showed that AR and 5αR2 expression was higher in all astrocytomas than the healthy brain; however, no statistically significant differences were observed between astrocytoma grades [110]. Werner and cols determined that AR expression at the transcript and protein levels in LN18, T98G glioblastoma cell lines, patient-derived xenografts (PDX), and human tumors overlapped with the expression of this receptor in primary prostate cancer. These authors also found that anti-androgens’ treatment slowed the growth and increased sensitivity to radiation of LN18, T98G, and U87 cell lines and patient-derived xenografts (in vitro and in vivo) [111]. These results suggest that T is involved in the growth of these tumors, regardless of the tumors' grade, in the progression toward a more proliferative, migratory, and invasive state (see Fig. 2). However, to establish an association between T and the prevalence of glioblastomas in men, AR and T levels in a larger group of biopsies from male and female glioblastoma patients must be measured.

## Conclusions

After compiling and analyzing the main actions of gonadal steroid hormones in glioblastoma cells, the more evident conclusion is that all of them can stimulate the progression of glioblastomas through the increase in proliferation, migration, and invasion. However, there are essential factors to consider since the effects mentioned above depend on the concentrations of hormones and the receptor involved in their actions. The actions exerted by female gonadal steroid hormones, E2 and P4, are the most controversial and complex. Depending on the type of receptor, ERα or ERβ, or hormone concentration, the actions of E2 and P4, respectively, have been associated with either a promoting or a protective role. Studies about T’s effects are less contradictory, and its role in glioblastoma progression has been broadly demonstrated by several authors in distinct models (see Fig. 2).

Regarding the role of gonadal steroid hormones in the prevalence of glioblastoma, the pieces of evidence found until the present is not sufficient to establish a conclusion. However, considering that they participate in the regulation of expression of several genes through their receptors, signaling pathways that have been associated with the prevalence of glioblastoma in both sexes could also be regulated by these steroids. The exogenous female gonadal steroid hormones consumed as hormonal contraceptives and hormone replacement therapy have been associated with a lower risk of developing glioblastoma, but more studies are required.

## Perspectives and significance

In general, this review highlights the urgency of finding more conclusive results about the role of gonadal steroid hormones in the progression and prevalence of glioblastomas, paying special attention to the differences between men and women. To answer this question, it is necessary to generate work models that allow the establishment of the differences between the concentration of hormones and the expression of their receptors in serum, tumor, and the tumor microenvironment in both sexes (see Figs. 1 and 2). These studies could be carried out in animal models, organotypic brain slice cultures, and patient biopsies. Complete knowledge in this area would establish an important precedent to determine the role of gonadal steroid hormones in the prevalence of glioblastomas and to establish more personalized and efficient therapies. Once the role of gonadal steroid hormones and their receptor in glioblastoma progression and prevalence have been completely demonstrated, new options for glioblastoma therapy could be incorporated, for example, agonists or antagonists of gonadal steroid hormones receptors, enzyme inhibitors, the gonadal steroid hormones themselves in concentrations effective to suppress tumor growth, and their genes and protein targets.

## Data Availability

Not applicable.

## Abbreviations

P4: Progesterone
E2: Estradiol
T: Testosterone
CNS: Central nervous system
TMZ: Temozolomide
EGFR: Epidermal growth factor receptor
ER: Estrogen receptor
PR: Progesterone receptor
AR: Androgen receptor
CDK: Cyclin-dependent kinase
TSGs: Tumor suppressor genes
GSC: Glioblastoma stem cell
DHP: Dihydroprogesterone
PAQR: Progestin and adipoQ receptor
mPRs: Membrane progesterone receptors
PGRMC 1-2: Progesterone receptor membrane component-1 and 2
DHT: 5α-dihydrotestosterone
5αR: 5α-reductase
PDX: Patient-derived xenografts

## References

[1] Louis DN, Perry A, Reifenberger G, von Deimling A, Figarella-Branger D, Cavenee WK, et al. The 2016 World Health Organization Classification of Tumors of the Central Nervous System: a summary. Vol. 131, Acta Neuropathologica. Springer Verlag; 2016. p. 803–820. 10.1007/s00401-016-1545-1 27157931

[2] Ostrom QT, Gittleman H, Truitt G, Boscia A, Kruchko C, Barnholtz-Sloan JS. CBTRUS statistical report: primary brain and other central nervous system tumors diagnosed in the United States in 2011-2015. Vol. 20, Neuro-Oncology. Oxford University Press; 2018. p. iv1–i86. 10.1093/neuonc/noy131 PMC6129949 30445539

[3] Kabat GC, Etgen AM, Rohan TE. Do steroid hormones play a role in the etiology of glioma? Vol. 19, Cancer Epidemiology Biomarkers and Prevention. American Association for Cancer Research; 2010. p. 2421–2427. 10.1158/1055-9965.EPI-10-0658 20841389

[4] Bhatia A, Sekhon HK, Kaur G. Sex hormones and immune dimorphism. Vol. 2014, Scientific World Journal. Hindawi Publishing Corporation; 2014.

[5] Siddiqui AN, Siddiqui N, Khan RA, Kalam A, Jabir NR, Kamal MA, Firoz CK, Tabrez S (2016). Neuroprotective role of steroidal sex hormones: an overview. CNS Neurosci Ther..

[6] Camacho-Arroyo I, Piña-Medina AG, Bello-Alvarez C, Zamora-Sánchez CJ. Sex hormones and proteins involved in brain plasticity. In: Gerald Litwack, editor. Vitamins and Hormones. Cambridge: Elsevier; 2020. p. 145–65. 10.1016/bs.vh.2020.04.002 32723542

[7] Rubin JB, Lagas JS, Broestl L, Sponagel J, Rockwell N, Rhee G, et al. Sex differences in cancer mechanisms. Vol. 11, Biology of Sex Differences. BioMed Central Ltd.; 2020. p. 17. 10.1186/s13293-020-00291-x PMC7161126 32295632

[8] González-Agüero G, Gutiérrez AA, González-Espinosa D, Solano JD, Morales R, González-Arenas A, Cabrera-Muñoz E, Camacho-Arroyo I (2007). Progesterone effects on cell growth of U373 and D54 human astrocytoma cell lines. Endocrine..

[9] Atif F, Yousuf S, Stein DG (2015). Anti-tumor effects of progesterone in human glioblastoma multiforme: Role of PI3K/Akt/mTOR signaling. J Steroid Biochem Mol Biol..

[10] Hernández-Hernández OT, González-García TK, Camacho-Arroyo I (2012). Progesterone receptor and SRC-1 participate in the regulation of VEGF, EGFR and Cyclin D1 expression in human astrocytoma cell lines. J Steroid Biochem Mol Biol..

[11] Atif F, Patel NR, Yousuf S, Stein DG. The synergistic effect of combination progesterone and temozolomide on human glioblastoma cells. PLoS One. 2015;10(6). 10.1371/journal.pone.0131441 PMC4482510 26110872

[12] Gutiérrez-Rodríguez A, Hansberg-Pastor V, Camacho-Arroyo I (2017). Proliferative and invasive effects of progesterone-induced blocking factor in human glioblastoma cells. Biomed Res Int..

[13] Altinoz MA, Ucal Y, Yilmaz MC, Kiris İ, Ozisik O, Sezerman U, Ozpinar A, Elmaci İ (2020). Progesterone at high doses reduces the growth of U87 and A172 glioblastoma cells: Proteomic changes regarding metabolism and immunity. Cancer Med..

[14] Piña-Medina AG, Hansberg-Pastor V, González-Arenas A, Cerbón M, Camacho-Arroyo I (2016). Progesterone promotes cell migration, invasion and cofilin activation in human astrocytoma cells. Steroids..

[15] Atif F, Yousuf S, Espinosa-Garcia C, Sergeeva E, Stein DG (2019). Progesterone treatment attenuates glycolytic metabolism and induces senescence in glioblastoma. Sci Rep..

[16] Piña-Medina AG, Díaz NF, Molina-Hernández A, Mancilla-Herrera I, Camacho-Arroyo I (2020). Effects of progesterone on the cell number of gliomaspheres derived from human glioblastoma cell lines. Life Sci..

[17] Zamora-Sánchez CJ, Hansberg-Pastor V, Salido-Guadarrama I, Rodríguez-Dorantes M, Camacho-Arroyo I (2017). Allopregnanolone promotes proliferation and differential gene expression in human glioblastoma cells. Steroids..

[18] Zamora-Sánchez CJ, Hernández-Vega AM, Gaona-Domínguez S, Rodríguez-Dorantes M, Camacho-Arroyo I (2020). 5alpha-dihydroprogesterone promotes proliferation and migration of human glioblastoma cells. Steroids..

[19] Germán-Castelán L, Manjarrez-Marmolejo J, González-Arenas A, González-Morán MG, Camacho-Arroyo I (2014). Progesterone induces the growth and infiltration of human astrocytoma cells implanted in the cerebral cortex of the rat. Biomed Res Int..

[20] Germán-Castelán L, Manjarrez-Marmolejo J, González-Arenas A, Camacho-Arroyo I (2016). Intracellular progesterone receptor mediates the increase in glioblastoma growth induced by progesterone in the rat brain. Arch Med Res..

[21] Cabrera-Muñoz E, González-Arenas A, Saqui-Salces M, Camacho J, Larrea F, García-Becerra R, Camacho-Arroyo I (2009). Regulation of progesterone receptor isoforms content in human astrocytoma cell lines. J Steroid Biochem Mol Biol..

[22] González-Arenas A, Hansberg-Pastor V, Hernández-Hernández OT, González-García TK, Henderson-Villalpando J, Lemus-Hernández D, Cruz-Barrios A, Rivas-Suárez M, Camacho-Arroyo I (2012). Estradiol increases cell growth in human astrocytoma cell lines through ERα activation and its interaction with SRC-1 and SRC-3 coactivators. Biochim Biophys Acta Mol Cell Res..

[23] Stupp R, Mason WP, van den Bent MJ, Weller M, Fisher B, Taphoorn MJB, Belanger K, Brandes AA, Marosi C, Bogdahn U, Curschmann J, Janzer RC, Ludwin SK, Gorlia T, Allgeier A, Lacombe D, Cairncross JG, Eisenhauer E, Mirimanoff RO (2005). Radiotherapy plus Concomitant and Adjuvant Temozolomide for Glioblastoma. N Engl J Med..

[24] Ober C, Loisel DA, Gilad Y. Sex-specific genetic architecture of human disease. Vol. 9, Nature Reviews Genetics. Nature Publishing Group; 2008. p. 911–922. 10.1038/nrg2415 PMC2694620 19002143

[25] Reinius B, Saetre P, Leonard JA, Blekhman R, Merino-Martinez R, Gilad Y (2008). An evolutionarily conserved sexual signature in the primate brain. Gibson G, editor. PLoS Genet.

[26] Yuan Y, Liu L, Chen H, Wang Y, Xu Y, Mao H, Li J, Mills GB, Shu Y, Li L, Liang H (2016). Comprehensive characterization of molecular differences in cancer between male and female patients. Cancer Cell..

[27] Kennelly R, Kavanagh DO, Hogan AM, Winter DC (2008). Oestrogen and the colon: potential mechanisms for cancer prevention. Lancet Oncol.

[28] Roshan MHK, Tambo A, Pace NP. The role of testosterone in colorectal carcinoma: Pathomechanisms and open questions. Vol. 7, EPMA Journal. BioMed Central Ltd.; 2016. 10.1186/s13167-016-0071-5 PMC5103431 27833666

[29] Ricketts CJ, Marston LW. Gender specific mutation incidence and survival associations in Clear Cell Renal Cell Carcinoma (CCRCC). PLoS One. 2015;10(10):e0140257. 10.1371/journal.pone.0140257 PMC4618848 26484545

[30] Chen KC, Lin CM, Huang CJ, Chen SK, Wu ST, Chiang HS, Ku WC (2016). Dual roles of 17-β estradiol in estrogen receptor-dependent growth inhibition in renal cell carcinoma. Cancer Genomic Proteomic..

[31] Pak S, Kim W, Kim Y, Song C, Ahn H (2019). Dihydrotestosterone promotes kidney cancer cell proliferation by activating the STAT5 pathway via androgen and glucocorticoid receptors. J Cancer Res Clin Oncol..

[32] Qin J, Liu M, Ding Q, Ji X, Hao Y, Wu X, Xiong J (2014). The direct effect of estrogen on cell viability and apoptosis in human gastric cancer cells. Mol Cell Biochem..

[33] Tang W, Liu R, Yan Y, Pan X, Wang M, Han X, Ren H, Zhang Z (2017). Expression of estrogen receptors and androgen receptor and their clinical significance in gastric cancer. Oncotarget..

[34] Kumar A, Klinge C, Goldstein R (2010). Estradiol-induced proliferation of papillary and follicular thyroid cancer cells is mediated by estrogen receptors α and β. Int J Oncol..

[35] Barakat R, Oakley O, Kim H, Jin J, Ko CMJ. Extra-gonadal sites of estrogen biosynthesis and function. Vol. 49, BMB Reports. The Biochemical Society of the Republic of Korea; 2016. p. 488–496. 10.5483/BMBRep.2016.49.9.141 PMC5227141 27530684

[36] Chowen JA, Torres-Aleman I, Garcia-Segura LM (1992). Trophic effects of estradiol on fetal rat hypothalamic neurons. Neuroendocrinology..

[37] Hernández-Fonseca K, Massieu L, Garca De La Cadena S, Guzmán C, Camacho-Arroyo I (2012). Neuroprotective role of estradiol against neuronal death induced by glucose deprivation in cultured rat hippocampal neurons. Neuroendocrinology..

[38] MacLusky NJ, Hajszan T, Prange-Kiel J, Leranth C (2006). Androgen modulation of hippocampal synaptic plasticity. Neuroscience..

[39] Sanchez AM, Flamini MI, Genazzani AR, Simoncini T (2013). Effects of progesterone and medroxyprogesterone on actin remodeling and neuronal spine formation. Mol Endocrinol..

[40] Guerra-Araiza C, Gómora-Arrati P, García-Juárez M, Armengual-Villegas A, Miranda-Martínez A, Lima-Hernández FJ (2009). Role of progesterone receptor isoforms in female sexual behavior induced by progestins in rats. Neuroendocrinology..

[41] Sun T, Warrington NM, Rubin JB (2012). Why does Jack, and not Jill, break his crown? Sex disparity in brain tumors. Biol Sex Differ..

[42] Ross JA, Davies SM (1999). Screening for neuroblastoma: progress and pitfalls - PubMed. Cancer Epidemiol Biomarkers Prev.

[43] Marinelli JP, Lohse CM, Carlson ML (2018). Incidence of Vestibular Schwannoma over the Past Half-Century: A Population-Based Study of Olmsted County, Minnesota. Otolaryngol Head Neck Surg (United States)..

[44] Lim JBT, Soeharno H, Tan MH. Sacral chordoma: clinical experience of a series of 11 patients over 18 years. Vol. 29, European Journal of Orthopaedic Surgery and Traumatology. Springer-Verlag France; 2019. p. 9–15. 10.1007/s00590-018-2284-x 30066091

[45] Sun T, Plutynski A, Ward S, Rubin JB. An integrative view on sex differences in brain tumors. Vol. 72, Cellular and Molecular Life Sciences. Birkhauser Verlag AG; 2015. p. 3323–3342. 10.1007/s00018-015-1930-2 PMC4531141 25985759

[46] Barabás K, Szegõ ÉM, Kaszás A, Nagy GM, Juhász GD, Ábrahám IM (2006). Sex differences in oestrogen-induced p44/42 MAPK phosphorylation in mouse brain in vivo. J Neuroendocrinol..

[47] Zhang L, Li B, Zhao W, Chang YH, Ma W, Dragan M (2002). Sex-related differences in MAPKs activation in rat astrocytes: Effects of estrogen on cell death. Mol Brain Res..

[48] Camacho-Arroyo I, Hansberg-Pastor V, Gutiérrez-Rodríguez A, Chávez-Jiménez J, González-Morán MG (2018). Expression of sex hormone receptors in the brain of male and female newly hatched chicks. Anim Reprod Sci..

[49] Matteoni S, Abbruzzese C, Villani V, Malorni W, Pace A, Matarrese P, et al. The influence of patient sex on clinical approaches to malignant glioma. Vol. 468, Cancer Letters. Elsevier Ireland Ltd; 2020. p. 41–47. 10.1016/j.canlet.2019.10.012 31605777

[50] Ohgaki H, Kleihues P. Genetic pathways to primary and secondary glioblastoma. Vol. 170, American Journal of Pathology. American Society for Investigative Pathology Inc.; 2007. p. 1445–1453. 10.2353/ajpath.2007.070011 PMC1854940 17456751

[51] Xu J, Khincha PP, Giri N, Alter BP, Savage SA, Wong JMY (2016). Investigation of chromosome X inactivation and clinical phenotypes in female carriers of DKC1 mutations. Am J Hematol..

[52] Jo EK, Kumaki S, Wei D, Tsuchiya S, Kanegane H, Song CH, Noh HY, Kim YO, Kim SY, Chung HY, Kim YH, Kook H (2004). X-linked Severe Combined Immunodeficiency Syndrome: The First Korean Case with γc Chain Gene Mutation and Subsequent Genetic Counseling. J Korean Med Sci..

[53] Dunford A, Weinstock DM, Savova V, Schumacher SE, Cleary JP, Yoda A, Sullivan TJ, Hess JM, Gimelbrant AA, Beroukhim R, Lawrence MS, Getz G, Lane AA (2017). Tumor-suppressor genes that escape from X-inactivation contribute to cancer sex bias. Nat Genet..

[54] Liau BB, Sievers C, Donohue LK, Gillespie SM, Flavahan WA, Miller TE (2017). Adaptive Chromatin Remodeling Drives Glioblastoma Stem Cell Plasticity and Drug Tolerance. Cell Stem Cell.

[55] Sun T, Warrington NM, Luo J, Brooks MD, Dahiya S, Snyder SC, Sengupta R, Rubin JB (2014). Sexually dimorphic RB inactivation underlies mesenchymal glioblastoma prevalence in males. J Clin Invest..

[56] Kfoury N, Sun T, Yu K, Rockwell N, Tinkum KL, Qi Z, Warrington NM, McDonald P, Roy A, Weir SJ, Mohila CA, Deneen B, Rubin JB (2018). Cooperative p16 and p21 action protects female astrocytes from transformation. Acta Neuropathol Commun..

[57] Altinoz MA, Ozpinar A, Elmaci I. Reproductive epidemiology of glial tumors may reveal novel treatments: high-dose progestins or progesterone antagonists as endocrino-immune modifiers against glioma. Vol. 42, Neurosurgical Review. Springer Verlag; 2019. p. 351–369. 10.1007/s10143-018-0953-1 29453736

[58] Batistatou A, Stefanou D, Goussia A, Arkoumani E, Papavassiliou AG, Agnantis NJ (2004). Estrogen receptor beta [ERβ] is espressed in brain astrocytic tumors and declines with dedifferentiation of the neoplasm. J Cancer Res Clin Oncol..

[59] Batistatou A, Kyzas PA, Goussia A, Arkoumani E, Voulgaris S, Polyzoidis K, Agnantis NJ, Stefanou D (2006). Estrogen receptor beta (ERβ) protein expression correlates with BAG-1 and prognosis in brain glial tumours. J Neurooncol..

[60] Sareddy GR, Nair BC, Gonugunta VK, Zhang QG, Brenner A, Brann DW, Tekmal RR, Vadlamudi RK (2012). Therapeutic significance of estrogen receptor β agonists in gliomas. Mol Cancer Ther..

[61] Li W, Winters A, Poteet E, Ryou MG, Lin S, Hao S, Wu Z, Yuan F, Hatanpaa KJ, Simpkins JW, Yang SH (2013). Involvement of estrogen receptor β5 in the progression of glioma. Brain Res..

[62] Verhaak RGW, Hoadley KA, Purdom E, Wang V, Qi Y, Wilkerson MD, Miller CR, Ding L, Golub T, Mesirov JP, Alexe G, Lawrence M, O'Kelly M, Tamayo P, Weir BA, Gabriel S, Winckler W, Gupta S, Jakkula L, Feiler HS, Hodgson JG, James CD, Sarkaria JN, Brennan C, Kahn A, Spellman PT, Wilson RK, Speed TP, Gray JW, Meyerson M, Getz G, Perou CM, Hayes DN, Cancer Genome Atlas Research Network (2010). Integrated genomic analysis identifies clinically relevant subtypes of glioblastoma characterized by abnormalities in PDGFRA, IDH1, EGFR, and NF1. Cancer Cell..

[63] Hernández-Vega AM, Del Moral-Morales A, Zamora-Sánchez CJ, Piña-Medina AG, González-Arenas A, Camacho-Arroyo I. Estradiol induces epithelial to mesenchymal transition of human glioblastoma cells. Cells. 2020;9(9):1930. 10.3390/cells9091930 PMC7564468 32825553

[64] Dueñas Jiménez JM, Candanedo Arellano A, Santerre A, Orozco Suárez S, Sandoval Sánchez H, Feria Romero I, López-Elizalde R, Alonso Venegas M, Netel B, de la Torre Valdovinos B, Dueñas Jiménez SH (2014). Aromatase and estrogen receptor alpha mRNA expression as prognostic biomarkers in patients with astrocytomas. J Neurooncol..

[65] Hönikl LS, Lämmer F, Gempt J, Meyer B, Schlegel J, Delbridge C (2020). High expression of estrogen receptor alpha and aromatase in glial tumor cells is associated with gender-independent survival benefits in glioblastoma patients. J Neurooncol..

[66] Paruthiyil S, Parmar H, Kerekatte V, Cunha GR, Firestone GL, Leitmant DC (2004). Estrogen receptor β inhibits human breast cancer cell proliferation and tumor formation by causing a G2 cell cycle arrest. Cancer Res..

[67] Yang W, Warrington NM, Taylor SJ, Whitmire P, Carrasco E, Singleton KW (2019). Sex differences in GBM revealed by analysis of patient imaging, transcriptome, and survival data. Sci Transl Med..

[68] Khalid H, Shibata S, Kishikawa M, Yasunaga A, Iseki M, Hiura T (1997). Immunohistochemical analysis of progesterone receptor and Ki-67 labeling index in astrocytic tumors - PubMed. Cancer..

[69] González-Agüero G, Ondarza R, Gamboa-Domínguez A, Cerbón MA, Camacho-Arroyo I (2001). Progesterone receptor isoforms expression pattern in human astrocytomas. Brain Res Bull..

[70] Hanada T, Rahayu TU, Yamahata H, Hirano H, Yoshioka T, Arita K (2016). Rapid malignant transformation of low-grade astrocytoma in a pregnant woman. J Obstet Gynaecol Res..

[71] Peeters S, Pagès M, Gauchotte G, Miquel C, Cartalat-Carel S, Guillamo JS, Capelle L, Delattre JY, Beauchesne P, Debouverie M, Fontaine D, Jouanneau E, Stecken J, Menei P, de Witte O, Colin P, Frappaz D, Lesimple T, Bauchet L, Lopes M, Bozec L, Moyal E, Deroulers C, Varlet P, Zanello M, Chretien F, Oppenheim C, Duffau H, Taillandier L, Pallud J (2018). Interactions between glioma and pregnancy: Insight from a 52-case multicenter series. J Neurosurg..

[72] Altinoz MA, Nalbantoglu J, Ozpinar A, Emin Ozcan M, Del Maestro RF, Elmaci I (2018). From epidemiology and neurodevelopment to antineoplasticity. Medroxyprogesterone reduces human glial tumor growth in vitro and C6 glioma in rat brain in vivo. Clin Neurol Neurosurg..

[73] Gutierrez-Rodriguez A, Camacho-Arroyo I (2016). Role of progesterone-induced blocking factor (PIBF) in pregnancy and cancer. TIP Rev Espec Ciencias Químico-biológicas..

[74] Check JH, Nazari P, Goldberg J, Yuen W, Angotti D (2001). A model for potential tumor immunotherapy based on knowledge of immune mechanisms responsible for spontaneous abortion. Med Hypotheses..

[75] Kyurkchiev D, Naydenov E, Tumangelova-Yuzeir K, Ivanova-Todorova E, Belemezova K, Bochev I, Minkin K, Mourdjeva M, Velikova T, Nachev S, Kyurkchiev S (2014). Cells isolated from human glioblastoma multiforme express progesterone-induced blocking factor (PIBF). Cell Mol Neurobiol..

[76] González-Arenas A, Valadez-Cosmes P, Jiménez-Arellano C, López-Sánchez M, Camacho-Arroyo I (2014). Progesterone-induced blocking factor is hormonally regulated in human astrocytoma cells, and increases their growth through the IL-4R/JAK1/STAT6 pathway. J Steroid Biochem Mol Biol.

[77] Check JH, Wilson C, Cohen R, Sarumi M (2014). Evidence that Mifepristone, a progesterone receptor antagonist, can cross the blood brain barrier and provide palliative benefits for glioblastoma multiforme grade IV - PubMed. Anticancer Res..

[78] Peluso JJ, Romak J, Liu X (2008). Progesterone receptor membrane component-1 (PGRMC1) is the mediator of progesterone’s antiapoptotic action in spontaneously immortalized granulosa cells as revealed by PGRMC1 small interfering ribonucleic acid treatment and functional analysis of PGRMC1 mutations. Endocrinology..

[79] Hlavaty J, Ertl R, Miller I, Gabriel C (2016). Expression of progesterone receptor membrane component 1 (PGRMC1), progestin and adipoQ receptor 7 (PAQPR7), and plasminogen activator inhibitor 1 RNA-binding protein (PAIRBP1) in glioma spheroids in vitro. Biomed Res Int..

[80] Valadez-Cosmes P, Vázquez-Martínez ER, Cerbón M, Camacho-Arroyo I. Membrane progesterone receptors in reproduction and cancer. Vol. 434, Molecular and Cellular Endocrinology. Elsevier Ireland Ltd; 2016. p. 166–175. 10.1016/j.mce.2016.06.027 27368976

[81] Valadez-Cosmes P, Germán-Castelán L, González-Arenas A, Velasco-Velázquez MA, Hansberg-Pastor V, Camacho-Arroyo I (2015). Expression and hormonal regulation of membrane progesterone receptors in human astrocytoma cells. J Steroid Biochem Mol Biol..

[82] Del Moral-Morales A, González-Orozco JC, Capetillo-Velázquez JM, Piña-Medina AG, Camacho-Arroyo I (2020). The role of mPRδ and mPRε in human glioblastoma cells: expression, hormonal regulation, and possible clinical outcome. Horm Cancer..

[83] González-Orozco JC, Hansberg-Pastor V, Valadez-Cosmes P, Nicolas-Ortega W, Bastida-Beristain Y, La Fuente-Granada MD (2018). Activation of membrane progesterone receptor-alpha increases proliferation, migration, and invasion of human glioblastoma cells. Mol Cell Endocrinol..

[84] Yague JG, Wang ACJ, Janssen WGM, Hof PR, Garcia-Segura LM, Azcoitia I, Morrison JH (2008). Aromatase distribution in the monkey temporal neocortex and hippocampus. Brain Res..

[85] Garcia-Segura LM, Wozniak A, Azcoitia I, Rodriguez JR, Hutchison RE, Hutchison JB (1999). Aromatase expression by astrocytes after brain injury: Implications for local estrogen formation in brain repair. Neuroscience..

[86] Pinacho-Garcia LM, Valdez RA, Navarrete A, Cabeza M, Segovia J, Romano MC (2020). The effect of finasteride and dutasteride on the synthesis of neurosteroids by glioblastoma cells. Steroids..

[87] Kretz O, Fester L, Wehrenberg U, Zhou L, Brauckmann S, Zhao S, Prange-Kiel J, Naumann T, Jarry H, Frotscher M, Rune GM (2004). Hippocampal synapses depend on hippocampal estrogen synthesis. J Neurosci..

[88] Mazzucco CA, Lieblich SE, Bingham BI, Williamson MA, Viau V, Galea LAM (2006). Both estrogen receptor α and estrogen receptor β agonists enhance cell proliferation in the dentate gyrus of adult female rats. Neuroscience..

[89] Farinetti A, Tomasi S, Foglio B, Ferraris A, Ponti G, Gotti S, Peretto P, Panzica GC (2015). Testosterone and estradiol differentially affect cell proliferation in the subventricular zone of young adult gonadectomized male and female rats. Neuroscience..

[90] Bao D, Cheng C, Lan X, Xing R, Chen Z, Zhao H, Sun J, Wang Y, Niu C, Zhang B, Fang S (2017). Regulation of p53wt glioma cell proliferation by androgen receptor-mediated inhibition of small VCP/p97-interacting protein expression. Oncotarget..

[91] Plunkett RJ, Lis A, Barone TA, Fronckowiak MD, Greenberg SJ (1999). Hormonal effects on glioblastoma multiforme in the nude rat model. J Neurosurg..

[92] Wigertz A, Lönn S, Mathiesen T, Ahlbom A, Hall P, Feychting M (2006). Risk of brain tumors associated with exposure to exogenous female sex hormones. Am J Epidemiol..

[93] Cowppli-Bony A, Bouvier G, Rué M, Loiseau H, Vital A, Lebailly P, Fabbro-Peray P, Baldi I (2011). Brain tumors and hormonal factors: review of the epidemiological literature. Cancer Causes Control..

[94] Qi Z-Y, Shao C, Zhang X, Hui G-Z, Wang Z (2013). Exogenous and endogenous hormones in relation to glioma in women: a meta-analysis of 11 case-control studies. Yang I, editor. PLoS One.

[95] Anic GM, Madden MH, Nabors LB, Olson JJ, Larocca RV, Thompson ZJ (2014). Reproductive factors and risk of primary brain tumors in women. J Neurooncol..

[96] Andersen L, Friis S, Hallas J, Ravn P, Kristensen BW, Gaist D (2015). Hormonal contraceptive use and risk of glioma among younger women: a nationwide case-control study. Br J Clin Pharmacol..

[97] Lan YL, Wang X, Lou JC, Ma BB, Xing JS, Zou S (2018). Update on the effect of exogenous hormone use on glioma risk in women: a meta-analysis of case-control and cohort studies. J Neurooncol..

[98] González-Arenas A, De la Fuente-Granada M, Camacho-Arroyo I, Zamora-Sánchez CJ, Piña-Medina AG, Segura-Uribe J (2019). Tibolone effects on human glioblastoma cell lines. Arch Med Res..

[99] Fernández-Guasti A, Kruijver FPM, Fodor M, Swaab DF (2000). Sex differences in the distribution of androgen receptors in the human hypothalamus. J Comp Neurol..

[100] Kruijver FPM, Fernández-Guasti A, Fodor M, Kraan EM, Swaab DF (2001). Sex differences in androgen receptors of the human mamillary bodies are related to endocrine status rather than to sexual orientation or transsexuality. J Clin Endocrinol Metab..

[101] Brentani MM, Lopes MTP, Martins VR, Plese JPP (1984). Steroid receptors in intracranial tumors. Clin Neuropharmacol..

[102] Paoletti P, Butti G, Zibera C, Scerrati M, Gibelli N, Roselli R, et al. Characteristics and biological role of steroid hormone receptors in neuroepithelial tumors. Journal of Neurosurgery Publishing Group. J Neurosurg. 1990;73(5):736–42. 10.3171/jns.1990.73.5.0736 2134312

[103] Stojković RR, Jovančević M, Jadro Šantel D, Grčević N, Gamulin S (1990). Sex steroid receptors in intracranial tumors. Cancer..

[104] Carroll RS, Zhang J, Dashner K, Sar M, Black PM (1995). Steroid hormone receptors in astrocytic neoplasms. Neurosurgery..

[105] Chung YG, Kim HK, Lee HK, Lee KC (1996). Expression of androgen receptors in astrocytoma. J Korean Med Sci..

[106] Liu C, Zhang Y, Zhang K, Bian C, Zhao Y, Zhang J (2014). Expression of estrogen receptors, androgen receptor and steroid receptor coactivator-3 is negatively correlated to the differentiation of astrocytic tumors. Cancer Epidemiol..

[107] Yu X, Jiang Y, Wei W, Cong P, Ding Y, Xiang L, Wu K (2015). Androgen receptor signaling regulates growth of glioblastoma multiforme in men. Tumor Biol..

[108] Zalcman N, Canello T, Ovadia H, Charbit H, Zelikovitch B, Mordechai A, Fellig Y, Rabani S, Shahar T, Lossos A, Lavon I (2018). Androgen receptor: a potential therapeutic target for glioblastoma. Oncotarget..

[109] Rodríguez-Lozano DC, Piña-Medina AG, Hansberg-Pastor V, Bello-Alvarez C, Camacho-Arroyo I (2019). Testosterone promotes glioblastoma cell proliferation, migration, and invasion through androgen receptor activation. Front Endocrinol (Lausanne).

[110] Rodríguez-Lozano DC, Velázquez-Vázquez DE, Del Moral-Morales A, Camacho-Arroyo I (2020). <p>Dihydrotestosterone Induces Proliferation, Migration, and Invasion of Human Glioblastoma Cell Lines</p>. Onco Targets Ther..

[111] Werner CK, Nna UJ, Sun H, Wilder-Romans K, Dresser J, Kothari AU, Zhou W, Yao Y, Rao A, Stallard S, Koschmann C, Bor T, Debinski W, Hegedus AM, Morgan MA, Venneti S, Baskin-Bey E, Spratt DE, Colman H, Sarkaria JN, Chinnaiyan AM, Eisner JR, Speers C, Lawrence TS, Strowd RE, Wahl DR (2020). Expression of the androgen receptor governs radiation resistance in a subset of glioblastomas vulnerable to antiandrogen therapy. Mol Cancer Ther..

